# Color expression in experimentally regrown feathers of an overwintering migratory bird: implications for signaling and seasonal interactions

**DOI:** 10.1002/ece3.994

**Published:** 2014-03-11

**Authors:** Christopher M Tonra, Kristen L D Marini, Peter P Marra, Ryan R Germain, Rebecca L Holberton, Matthew W Reudink

**Affiliations:** 1 Migratory Bird Center, Smithsonian Conservation Biology InstituteNational Zoological Park, Washington, District of Columbia; 2 School of Biology and Ecology, University of MaineOrono, Maine; 3 Department of Biological Sciences, Thompson Rivers UniversityKamloops, BC V2C 0C8, Canada; 4 Centre for Applied Conservation Research, University of British ColumbiaVancouver, BC V6T 1Z4, Canada

**Keywords:** American redstart, carotenoid, delayed plumage maturation, molt, plumage color, *Setophaga ruticilla*

## Abstract

Plumage coloration in birds plays a critical role in communication and can be under selection throughout the annual cycle as a sexual and social signal. However, for migratory birds, little is known about the acquisition and maintenance of colorful plumage during the nonbreeding period. Winter habitat could influence the quality of colorful plumage, ultimately carrying over to influence sexual selection and social interactions during the breeding period. In addition to the annual growth of colorful feathers, feather loss from agonistic interactions or predator avoidance could require birds to replace colorful feathers in winter or experience plumage degradation. We hypothesized that conditions on the wintering grounds of migratory birds influence the quality of colorful plumage. We predicted that the quality of American redstart (*Setophaga ruticilla*) tail feathers regrown after experimental removal in Jamaica, West Indies, would be positively associated with habitat quality, body condition, and testosterone. Both yearling (SY) and adult (ASY) males regrew feathers with lower red chroma, suggesting reduced carotenoid content. While we did not observe a change in hue in ASY males, SY males shifted from yellow to orange plumage resembling experimentally regrown ASY feathers. We did not observe any effects of habitat, testosterone, or mass change. Our results demonstrate that redstarts are limited in their ability to adequately replace colorful plumage, regardless of habitat, in winter. Thus, feather loss on the nonbreeding grounds can affect social signals, potentially negatively carrying over to the breeding period.

## Introduction

Visual signals in animals can play an essential role in an individual's fitness (Maynard Smith and Harper 2003). Ornaments, such as colorful plumage, can serve as honest signals of individual quality which function in both inter- (Kodric-Brown and Brown 1984; Hill 2006) and intrasexual communication (Maynard Smith and Harper 1988; McGraw and Hill 2000) and thus may be under selection across different phases of the annual cycle. Therefore, developing high-quality ornaments and maintaining their quality throughout the year may be critically important to maximizing fitness. For colorful plumage limited by the environmental availability of pigments (e.g., carotenoids; Hill 1992), social factors (e.g., social dominance; Karubian et al. 2011; Maia et al. 2012), or physical condition (e.g., Saks et al. 2003; Barron et al. 2013), dominant individuals in high-quality habitats should have an advantage in producing and maintaining high-quality plumage. In addition, the acquisition of high-quality plumage may be influenced by the timing and duration of molt (Griggio et al. 2009; Newton and Dawson 2011; Stutchbury et al. 2011).

In migratory birds, the quality of nonbreeding (stationary and migratory) habitats may be inextricably linked to breeding success through limitation of signal quality. Nonbreeding season conditions can carry over to influence multiple aspects of migration and breeding season performance (e.g., Bearhop et al. 2004; Gunnarsson et al. 2005; Reudink et al. 2009a; Tonra et al. 2011). Revealing such seasonal interactions (Marra et al. 1998; Runge and Marra 2005) is critical to understanding the ecology of migratory birds. However, no study to date has examined how stationary nonbreeding (hereafter: winter) habitat effects on spectral signal production may carry over to influence breeding success (but see Saino et al. 2004; Sorensen et al. 2010 for studies of ornament size).

In passerines, the vast majority of species in the Western Hemisphere undergo a single annual complete molt, generally during the postbreeding period while still near the breeding grounds (Pyle 1997; Gill 2006). Many of these feathers serve as important ornaments that play a role in breeding success and therefore must be maintained though daily maintenance (e.g., preening; Griggio et al. 2010) and by adventitiously replacing lost feathers while occupying varying habitats through the year. Winter habitats may influence plumage quality either directly or indirectly. For carotenoid-based coloration, the dietary availability of carotenoid pigments within the habitat used by an individual during molt (adventitious or obligate) should directly limit the pigment concentration in feathers (e.g., Hill 1992). Indirectly, food availability within a habitat may limit body condition (e.g., Studds and Marra 2005) which could in turn limit plumage quality (e.g., Hill and Montgomerie 1994; Barron et al. 2013). If the social environment varies among habitats, this could in turn mediate the acquisition, development, and/or expression of ornamental traits (Karubian et al. 2011; Maia et al. 2012), perhaps due to increased rates of feather loss where aggressive interactions are frequent. Finally, habitat effects on hormones that can influence carotenoid availability, such as testosterone (hereafter: T) in males, may indirectly influence plumage quality (e.g., Blâs et al. 2007; but see Barron et al. 2013). Elucidating these relationships requires observing the growth of colorful feathers in different winter habitats within individuals in variable condition, and measuring effects on resulting coloration.

**Figure 1:**
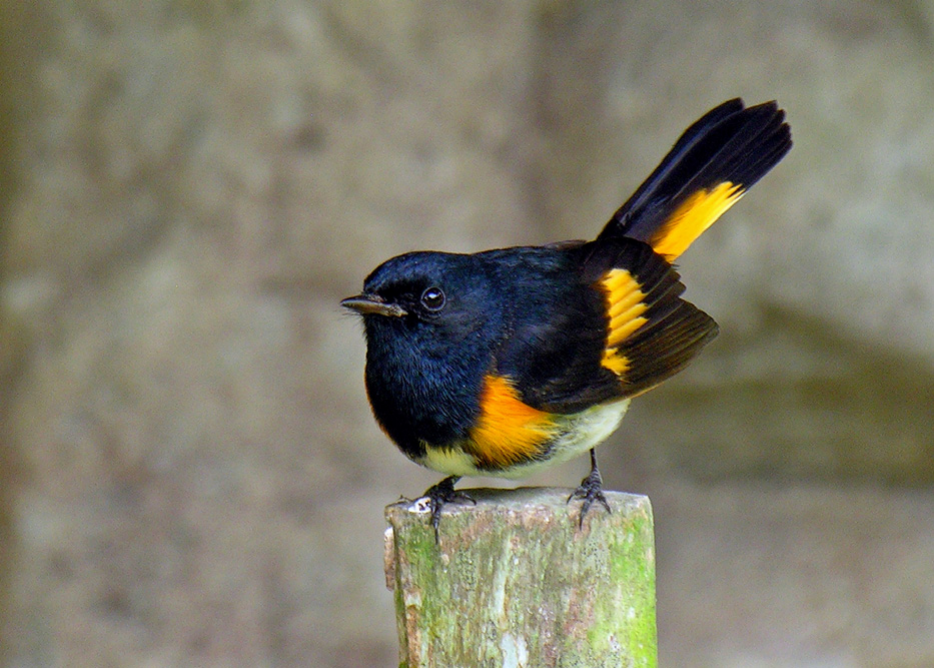
An after second year (ASY) male American redstart (*Setophaga ruticilla*; © Dennis Jarvis).

The American redstart (*Setophaga ruticilla*) is a migratory songbird that overwinters in the neotropics and is territorial during the winter period (Sherry and Holmes 1997). Compared with individuals in low-quality dry habitats, birds that winter in high-quality wet habitats maintain body mass (Marra and Holberton 1998; Studds and Marra 2007) and depart earlier for spring migration (Marra et al. 1998; Studds and Marra 2005). Male redstarts from high-quality winter habitats arrive earlier at the breeding grounds (Marra et al. 1998; Reudink et al. 2009a; Tonra et al. 2011) with higher circulating androgens (Tonra et al. 2011) and ultimately sire more offspring (Reudink et al. 2009a) than those from low-quality habitats. Male redstarts have colorful, carotenoid-based plumage on their flanks and tails (Fig. 1). The brightness and redness of these areas predict parental investment (Germain et al. 2010a) and breeding success (Reudink et al. 2009b,c). Offspring of males with brighter flanks are fed more often (Germain et al. 2010a). Males with brighter tails are more likely to be polygynous, and those with redder flanks are less likely to lose paternity (Reudink et al. 2009b). Furthermore, redstarts arriving to breed from more mesic winter habitats have brighter tails (Reudink et al. 2009c), which may indicate behavioral dominance (Marra 2000). Redstarts undergo a single, complete obligate molt (Sherry and Holmes 1997), but must often replace feathers adventitiously during the winter period. In winter, 10–22% of redstarts captured annually experience tail feather loss and replacement, (2008–2010; P. P. Marra and C. M. Tonra unpubl. data), and redstarts show increasing evidence of body feather replacement as winter progresses (Rohwer et al. 1983). Replacement of lost feathers is more likely in individuals that maintain or gain mass (Reudink et al. 2008), but the role of condition in the replaced feather quality is unknown. The redstart system provides an ideal opportunity to examine how winter conditions can influence plumage integral to breeding success.

An additional level of complexity in this system is the existence of delayed plumage maturation in male redstarts (Rohwer et al. 1980; Germain et al. 2012). Yearling (second year, hereafter: SY) males resemble females (gray and yellow plumage), but exhibit some small patches of mature male plumage (black and orange plumage) to varying extents (Sherry and Holmes 1997; Germain et al. 2010b). SY males that exhibit more adult (after second year, hereafter: ASY)-like melanin-based plumage are more likely to occupy high-quality winter territories and arrive earlier at breeding sites (Germain et al. 2010b). In this way, the extent of ASY-like plumage in yearling males may be an honest signal of individual quality, potentially influencing the ability of birds to acquire breeding territories in their first breeding season. The extent to which winter habitat, body condition, and T may influence the acquisition of ASY-like carotenoid-based plumage is not currently known.

Here, we examine the influence of winter habitat quality, testosterone, and condition on the production and maintenance of colorful traits that are associated with breeding success in a migratory bird. We experimentally removed and collected a replaced tail feather from male redstarts overwintering in Jamaica, West Indies, to test the hypothesis that winter habitat quality influences plumage quality either directly or through a condition-mediated mechanism. We predicted that ASY males in high-quality mangrove habitat would regrow more colorful feathers (higher red chroma, more orange-shifted hue) than those in low-quality second-growth scrub. Furthermore, we predicted that mass change and circulating T would be positively related to feather coloration. Lastly, we examined these relationships in yearling males, testing the prediction that more ASY-like (i.e., orange-shifted hue) replacement feathers would be positively related to habitat quality, mass change, and T.

## Methods

### Field work

Field work took place between January and May, 2008–2010, with a long-term study population of American redstarts at the Font Hill Nature Preserve (18°02′N, 77°57′W), St. Elizabeth Parish, Jamaica. Male redstarts were studied in two habitat types: high-quality mangrove habitat dominated by black mangrove (*Avicennia germinans*) and lower-quality second-growth scrub habitat consisting primarily of logwood (*Haematoxylon campechianum*; see Marra 2000; Studds and Marra 2005 for a complete description). Upon initial capture in January to March, each male (*n* = 65) was given a unique combination of a USGS aluminum band and two plastic color bands. Each bird was then weighed, and a single rectrix (R3) was removed from the tail before it was released. Each male was aged as SY or ASY based on plumage color (Pyle 1997). To establish territory boundaries, and thus validate habitat use, each banded male's territory was mapped throughout the mid- to late-winter period (Jan–May). Individuals were then recaptured at least a month after the initial capture, to allow time for the growth of an induced feather (Grubb and Cimprich 1990), which was plucked upon recapture of the individual. To test whether overwinter mass change, an index of body condition in overwintering redstarts (e.g., Studds and Marra 2005), predicted regrown feather color, and whether this change was dependent on winter habitat, we weighed each individual to the nearest 0.1 g on both occasions. Mass change was recorded as final weight – initial weight, with positive values indicating overwinter mass gain. For a subset of SY males captured from both habitats during their hatch year in fall (Oct–Nov) 2008 (*n* = 17), we took 4–5 standardized photographs of each individual following Germain et al. (2010b) and alternately assigned each SY to either an experimental or control group. We plucked 15–20 gray (SY-like) feathers from the breast of each individual in the experimental group and plucked no feathers from control birds. The following spring (mid-late January 2009), we recaptured individuals from each group and took an additional 4–5 photographs to determine the extent of ASY-like feather regrowth. Due to a low recapture rate of experimental and control birds in spring, we captured two additional unmanipulated SY males in spring to provide a further baseline comparison against feather regrowth in experimentally plucked individuals.

### Color analysis

Plumage color was quantified by measuring reflectance across the avian visual range (300–700 nm) using an Ocean Optics JAZ spectrometer (Dunedin, FL) with a PX-2 xenon light source. Light was transmitted through a fiber optic probe held at a 90° angle by a nonreflective probe holder to consistently measure the feather from a set distance of 5.9 mm. To standardize the reflectance measurements, dark (sealed cylinder of Colorline #142 Ebony paper) and white (Ocean Optics white standard) standards were used to calibrate the spectrometer between each feather (*n* = 140). Each feather was mounted on low-reflectance black paper, and ten measurements were taken from the yellow-orange patch of each feather. Feathers with insufficient carotenoid color, where the colored area was too small to accurately measure, were not included in color analysis.

Reflectance data were analyzed using the R-based RCLR 0.9.29 color analysis program (Kingston, ON, Canada, http://post.queensu.ca/˜mont/color/) to calculate three color variables: brightness, red chroma, and hue (Montgomerie 2008). Brightness (mean *R*300-700) was measured as the mean amount of light reflected across all wavelengths (300–700 nm). Red chroma was calculated as: *R*605-700/*R*300-700 as a measure of spectral purity, measuring the amount of light reflected in the red-orange region of the spectrum relative to the entire spectrum. Hue was calculated as: arctan ([(*R*415-510–*R*320-415)/*R*320-700]/[(*R*575-700–*R*415-575)/*R*320-700]) and provides information on the dominant wavelength of light reflected by the feather, measured as a ratio of light reflected by different segments in the spectrum.

To measure the extent of ASY-like melanin-based plumage in SY males, we uploaded all standardized photographs into Adobe Photoshop CS3 (v 10.0) following Germain et al. 2010b and used the lasso tool to measure the area of black (ASY-like) plumage on the chin, throat, and breast (hereafter called “breast”). We then standardized the total area of black breast plumage for each individual by wing length (mm) to control for body size (Germain et al. 2010b).

### Testosterone analysis

In a subset of birds (*n* = 24), we measured circulating testosterone (T) in blood samples taken on both capture occasions from 2009 to 2010. We collected a 50–80 *μ*L blood sample within 5 min of our approach to the net and stored it as described by Tonra et al. (2013). We have examined the effects of different capture method (i.e., passive netting, where birds may spend more time in the net, versus use of conspecific playback, where birds are immediately removed) on circulating T in this population and not found any effects (Tonra et al. 2013). To determine plasma androgen concentration, we used a direct radioimmunoassay (Wingfield et al. 1992) for T. We ran separate assays for each year's samples, run in duplicate, but all samples collected within a year were run in a single assay. Interassay percent coefficient of variation, based on a commercial standard, was 10%. Intra-assay variation was 5% and 4%, in 2009 and 2010, respectively. The sensitivity of the standard curve in both years was 1.7 pg/mL. The T antibody (T3-125, Endocrine Sciences/Esoterix®, Calabasas, CA) has high specificity for T but also at least 44% cross-reactivity with dihydrotestosterone, which parallels patterns of seasonal change in T (Wingfield and Farner 1978).

### Statistical analysis

All statistical analyses were conducted in JMP version 10 (SAS Institute Inc.) and R version 3.0 (R Core Development Team 2013). We first used analysis of variance (ANOVA) or Student's *t*-tests to examine potential year effects for all predictor and response variables and found no significant differences in T (*t*_*24*_ = −0.78, *P* = 0.45), or original or regrown feather color variables among ASY males (all *P* > 0.18). Unequal variance between years, due to small sample sizes in 2008 (*n* = 4) and 2010 (*n* = 3) precluded testing for year effects on SY male color variables. We did find significant differences among years in overwinter mass change (*F*_2,60_ = 5.72, *P* = 0.005); we thus standardize overwinter mass change by year setting the mean to 0 with a standard deviation of 1. Testosterone data were only available for one year. We used Student's *t-*tests to describe color differences between SY and ASY males. Next, we examined changes in color between original and regrown feathers using paired *t*-tests. Because some individuals were repeated across years, we used linear mixed models with individual as a random effect to examine the effect of habitat (mangrove/logwood) on original feather color, regrown feather color, and the difference between original and regrown feather color (i.e., magnitude of color change) using the lmer function in R. To assess the significance of each variable in mixed models, we iteratively removed it from the model and compared the reduced model to a model of only the main effects (for testing main effects) or to the full model (for testing interactions) using a likelihood ratio test (Zurr et al. 2009). To examine whether mass change predicted color, we used Pearson correlations with SY males and linear mixed models with ASY males. In addition, we built models using habitat and mass change and a habitat by mass change interaction term to test for the habitat effects on the relationship between these independent variables and color. Finally, to examine the relationships between recapture T (log-transformed), T change, and color, we used Pearson correlations (no ASY males were repeated in this analysis).

### Ethics statement

All animal research activities were approved by the University of Maine Institutional Animal Care and Use Committee (protocols A2006-07-04 and A2009-06-05). These activities were conducted in accordance with a federal bird banding permit from the United States Geological Survey and a research permit from the National Environmental Protection Agency of Jamaica, both held by Marra.

## Results

### Age-related differences in color

**Figure 2:**
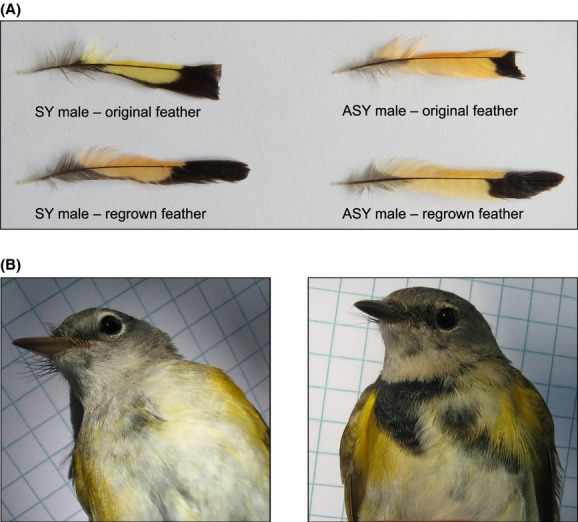
Photographs of original and regrown feathers. A) Photographs of SY and ASY male tail feathers illustrating the color change between original and regrown feathers. SY males experienced a significant reduction in red chroma and shift in hue toward ASY-like orange coloration. ASY males displayed a reduction in brightness and red chroma, but no significant change in hue. B) Photographs of an SY male prior to breast feather plucking (left) and after feather regrowth (right).

**Figure 3:**
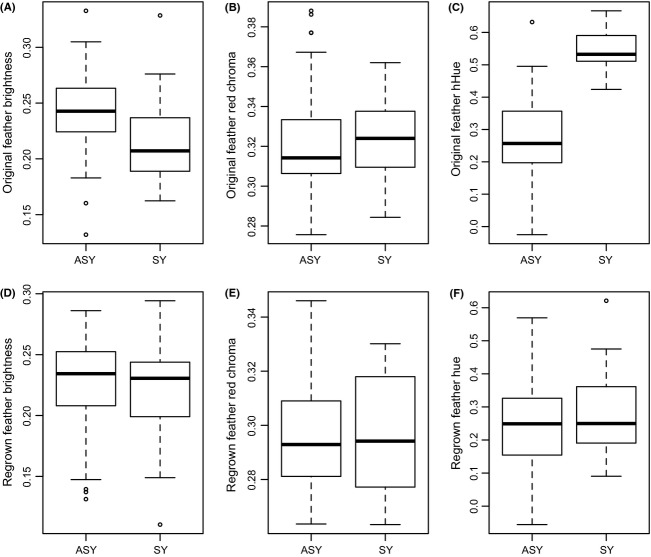
Age-related differences in feather color. Originally grown feathers of ASY males (*n* = 43) are significantly brighter than SY males (*n* = 22) (A) and have significantly more negative hue values (C), shifted more toward the orange/red region of the visual spectrum. There was no difference between ASYs and SYs in red chroma (B). Regrown feathers did not differ in brightness (D), chroma (E), or hue (F) between ASY and SY males.

When we quantified the differences in color between age classes in the yellow/orange region of originally grown tail feathers, ASY (*n* = 43) and SY (*n* = 22) male feathers differed in hue, with ASY males exhibiting lower values, indicative of longer (red/orange) wavelengths (*t *=* *11.08, *P *<* *0.0001; Figs. 2A, 3). ASY males exhibited higher brightness values than SY males (*t *=* *−2.74, *P *=* *0.008), but no differences in red chroma were evident (*t *=* *0.24, *P *=* *0.81). Upon recapture, when we examined the color of regrown feathers, we found no differences in hue (*t *=* *1.20, *P *=* *0.23), brightness (*t *=* *−0.31, *P *=* *0.75), or red chroma (*t *=* *−0.06, *P *=* *0.95) between age classes (Figs. 2A, 3).

### Changes in color from original to regrown feathers

**Figure 4:**
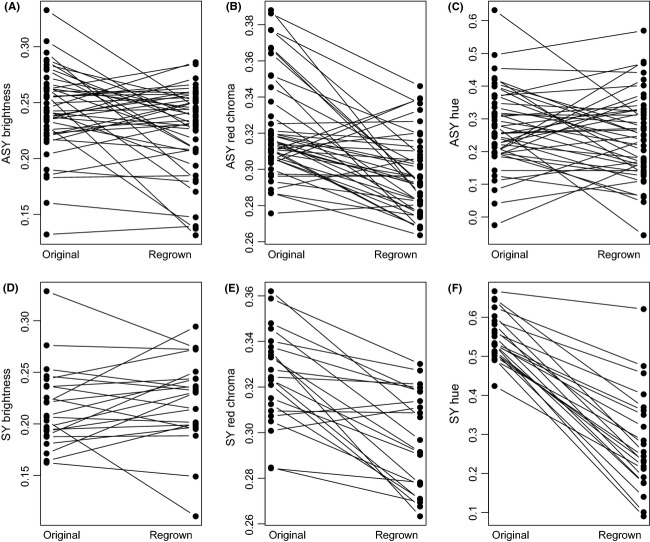
Differences between original and regrown feather color. ASY males (top; *n* = 43) exhibited a significant reduction in brightness (A) and red chroma (B), but no change in hue (C) between original and regrown feathers. SY males (bottom; *n* = 22) exhibited a significant reduction in chroma (E) and shift in hue (F) from yellow to more ASY-like orange, but no change in brightness (D).

When we examined changes in feather color from original to regrown feathers, we found that ASY (*n* = 43) males exhibited a decrease in brightness (original: 0.243 ± 0.04 SD, regrown: 0.225 ± 0.04 SD, *t *=* *−2.70, *P *=* *0.01), a decrease in red chroma (original: 0.322 ± 0.03 SD, regrown: 0.297 ± 0.02 SD, *t *=* *−5.51, *P *<* *0.0001), but no change in hue (original: 0.273 ± 0.12 SD, regrown: 0.245 ± 0.13 SD, *t *=* *−1.31, *P *=* *0.20; Figs. 2A, 4). SY males (*n* = 22) exhibited no change in brightness (original: 0.216 ± 0.04 SD, regrown: 0.222 ± 0.04 SD, *t *=* *0.77, *P *=* *0.45), but a reduction in chroma (original: 0.324 ± 0.02 SD, regrown: 0.296 ± 0.02 SD, *t *=* *−5.73, *P *<* *0.0001) and a decrease in hue values consistent with a shift toward ASY-like orange feathers (original: 0.552 ± 0.06 SD, regrown: 0.281 ± 0.13 SD, *n* = 22, *t *=* *−11.94, *P *<* *0.0001; Figs. 2A, 4).

**Figure 5:**
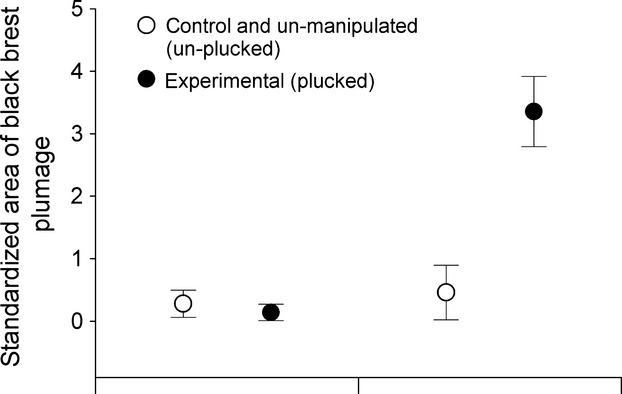
Changes in the extent of black plumage in SY males. Mean (±SD) area (mm^2^) of black breast plumage (standardized by wing length) for SY males captured in Fall 2008 and Spring 2009. Individuals experimentally plucked and recaptured the next season (*n* = 4) exhibited substantial increases in the extent of black breast patches, compared with control birds captured in fall (*n* = 13) and spring (*n* = 1), as well as two unmanipulated birds captured in spring only, each of which had no experimentally plucked feathers. One control bird originally banded in fall and recaptured in spring exhibited no change in the extent of black breast plumage.

For black (melanin-based) plumage regrowth in SY males, we recaptured one control bird and four birds that were experimentally plucked in fall (Figs. 2B, 5). The extent of black breast plumage from experimental birds (*n* = 2 in mangrove, two in scrub) showed between a 10×-107× increase in black breast plumage overall (Figs. 2B, 5). The one control recapture (mangrove) exhibited virtually no change in black breast plumage (0.49 fall, 0.39 spring). In addition, all experimental birds exhibited markedly more ASY-like black plumage than two subsequent unmanipulated birds caught in spring but not in fall (0.05 and 0.9, respectively; Figs. 2B, 5).

### Color and habitat

We found no differences in any color variables of original feathers between birds living in mangrove versus logwood habitat, in either ASY or SY males (all *P *>* *0.33). Similarly, there were no differences in color in regrown feathers (all *P *>* *0.28) between habitats.

### Color and testosterone

We collected repeated T samples from 11 SY and 13 ASY male redstarts. We found no relationships between regrown feather color and recapture T in ASY (brightness: *r *=* *0.06, *P *=* *0.70; red chroma: *r *=* *−0.24, *P *=* *0.44; hue: *r *=* *−0.20, *P *=* *0.52) or SY (brightness: *r *=* *−0.16, *P *=* *0.64, red chroma: *r *=* *−0.14, *P* = 0.68, hue: *r *=* *0.13, *P *=* *0.71) male redstarts. Nor did we find any relationships between T change and change in feather brightness (ASY: *r *=* *0.05, *P *=* *0.87; SY: *r *=* *0.08, *P *=* *0.81), red chroma (ASY: *r *=* *0.33, *P *=* *0.27; SY: *r *=* *−0.38, *P *=* *0.25), or hue (ASY: *r *=* *−0.26, *P *=* *0.39; SY: *r* = −0.05, *P *=* *0.89).

### Color and condition

Mass change did not predict regrown feather color in ASY males (*n* = 41, all *P *>* *0.62), nor were there any mass change*habitat interactions (all *P *>* *0.38); however, mass change was negatively correlated with red chroma in SY males (i.e., birds that lost mass overwinter had higher red chroma values), with no mass change*habitat interaction (mass change: *n* = 21, *r *=* *0.55, *P *=* *0.01; habitat*mass change: *n* = 21, *F*_1,19_ = 0.34, *P *=* *0.57), and no relationship with brightness or hue (both *P *>* *0.31, interactions: *P *>* *0.10).

## Discussion

We predicted that habitat quality, T, and energetic condition would be positively related to the quality of colorful feathers. In studies focused on the breeding season, these factors have been known to influence feather color (e.g., Hill and Montgomerie 1994; Blâs et al. 2007; Ferns and Hinsley 2008; Lindsay et al. 2011; Barron et al. 2013). Here, however, we experimentally induced colorful plumage production in a wintering migratory bird and failed to find support for these predictors. Furthermore, we found that the quality of colorful feathers is diminished when replaced on the wintering grounds, particularly for mature males. Our findings suggest that 1) condition-mediated mechanisms for plumage quality may not be operating in winter and 2) there is a significant cost to plumage quality when feathers are lost in winter. Furthermore, we suggest that plumage maturation in young males may be accelerated by events that induce feather replacement.

Both ASY and SY males exhibited reductions in red chroma in regrown feathers. Chroma correlates closely to feather carotenoid concentration (Saks et al. 2003) and reflects dietary carotenoid availability, individual foraging ability, and/or condition. Thus, regardless of age, habitat, testosterone, or condition, a reduction in red chroma suggests that all males regrowing feathers on the wintering grounds appear to incorporate fewer carotenoids in regrown feathers. If red chroma is indeed reflecting low concentrations of carotenoids, this could suggest that either a) carotenoid availability on the wintering grounds in Jamaica is limited or b) individuals are physiologically limited in their ability to utilize carotenoids for functions other than maintenance. Based on our results, we suggest that physiological limitation is unlikely, as we did not observe any relationships between T, condition, or habitat quality and the color of regrown feathers. However, one possibility is that environmental conditions during the years studied (2008–2010) were not extreme enough to produce habitat-specific differences strong enough to impact feather color. We suggest that the most likely explanation for the reduction in red chroma observed in both ASY and SY males is due to limitation of dietary carotenoids. While redstarts consume large numbers of carotenoid-rich lepidopteron prey (Robinson and Holmes 1982; Sherry and Holmes 1997; Eeva et al. 2010) on the breeding grounds, they consume fewer lepidopteron prey in the winter (Sherry and Holmes 1997), suggesting a dietary mechanism for the observed degradation in feather color.

Although we did not observe any changes in brightness between original and regrown feathers in SY males, we did observe a significant reduction in brightness in ASY males. Previous work on redstarts found that tail brightness was positively associated with both overwinter habitat quality (a pattern we did not observe in this study; Reudink et al. 2009c) and polygyny during the breeding season (Reudink et al. 2009b). Thus, a reduction in tail brightness in adventitiously molted feathers could potentially have negative consequences for territory acquisition or polygynous mating. Whether the loss and regrowth of a single feather (or several feathers) is enough to alter the signal content for potential receivers remains untested. However, given that 10–22% of males in this study population (based on banding records from 2008 to 2010; Marra and Tonra unpubl. data) were observed missing or replacing one or more tail feathers, the loss and regrowth of feathers during the nonbreeding period has the potential to impact signals important for reproduction. Furthermore, replacement of color in tail feathers can be viewed as a proxy for replacement of the same colors in body plumage, such as flanks, which are important to sexual selection (Reudink et al. 2009b; Germain et al. 2010a), and subject to loss in winter (Rohwer et al. 1983).

When we examined changes in hue among nonbreeding redstarts, we found age class-specific differences. ASY males did not experience a change in hue (although visually a change in the appearance of regrown feathers is clearly evident (e.g., Fig 2A) and reflected by a significant reduction in red chroma). In contrast, SY males experienced a significant reduction in hue between original and regrown feathers, indicating a shift from the typical SY yellow coloration to more ASY-like orange coloration. In ASY feathers, orange coloration is produced from the deposition of two yellow carotenoid pigments (canary xanthophyll A, canary xanthophyll B) and a red-orange pigment (canthaxanthin), which is produced from the metabolic conversion of beta-carotene (McGraw et al. 2004; McGraw 2006). Although carotenoid content of SY American redstart feathers has not been examined to our knowledge, it is likely that SY feathers contain only canary xanthophylls A and B and do not contain canthaxanthin. Interestingly, however, our data suggest that after the initial growth of SY plumage (grown during the nestling/fledgling phase; Pyle 1997; Sherry and Holmes 1997), a physiological shift occurs. This shift enables SYs to either synthesize canthaxanthin or, if canthaxanthin was already being metabolized from beta-carotene, afford to utilize canthaxanthin in feather production rather than maintenance. Further evidence for this physiological shift comes from our data on regrown body feathers. Although our sample size is limited, each recaptured SY male that had breast feathers plucked on the nonbreeding grounds in the fall regrew ASY-typical black plumage in its place, leading to more extensive ASY-like plumage overall. Although only one control (nonplucked) SY male was recaptured in spring, this male exhibited no change in the extent of ASY-like black plumage and was representative of all other unmanipulated (nonplucked) SYs captured in spring. Previously, Germain et al. (2010b) found that SY males occupying high-quality mangrove habitat had significantly more extensive adult-like black plumage on their breast. Given that the incidence of melanin-based ASY-like plumage in SY birds increases as winter progresses (Rohwer et al. 1983), the authors suggest this habitat pattern is likely indicative of feather loss through agonistic interactions, as occupancy of high-quality mangrove habitat appears is driven primarily by dominance interactions (Marra 2000). Because melanin-based black plumage may be hormonally controlled (reviewed in McGraw 2006), our results further the notion that young males undergo a physiological shift as they age. This suggests that the selective pressures resulting in female-like plumage are operating more strongly in the beginning than the end of the nonbreeding season.

In conclusion, adult American redstart males are constrained in winter in their ability to regrow feathers with color similar to their original feathers grown near breeding areas. This color difference is likely due to either a lack of available dietary carotenoids or an inability to utilize available carotenoids for feather production (e.g., if carotenoids are needed for immune system maintenance). Given the importance of these color patches to reproductive success (e.g., Reudink et al. 2009b), a change in color characteristics due to plumage growth outside of the normal period of molt could be a significant cost of feather loss that carries over to negatively impact breeding success, which we suggest warrants future study. In an evolutionary sense, these findings could suggest winter limitation of the ability to acquire colorful plumage as an explanation for the maintenance of a single annual molt in redstarts, while most *Setophaga* replace colorful feathers on the wintering grounds during a second pre-alternate molt (Pyle 1997). Alternatively, interspecific variation in plumage coloration may be explained by environmental constraints resulting from the timing and location of molt (e.g., a single postbreeding molt near the breeding grounds or an additional pre-alternate molt on the wintering grounds). Future comparative studies that examine the relationships among molt strategies, plumage coloration, and ecological constraints (e.g., carotenoid availability) will be extremely useful for understanding the evolution of molt strategies and plumage coloration across Setophaga and other Neotropical migrants.
